# 3-{5-Eth­oxy­carbonyl-1-[3-(2-oxopyrrol­idin-1-yl)prop­yl]-1*H*-benzimidazol-2-yl}benzoic acid

**DOI:** 10.1107/S1600536813001116

**Published:** 2013-01-31

**Authors:** Yeong Keng Yoon, Mohamed Ashraf Ali, Soo Choon Tan, Mohd Mustaqim Rosli, Ibrahim Abdul Razak

**Affiliations:** a Institute for Research in Molecular Medicine, Universiti Sains Malaysia, 11800 USM, Penang, Malaysia; b X-ray Crystallography Unit, School of Physics, Universiti Sains Malaysia, 11800 USM, Penang, Malaysia

## Abstract

In the title compound, C_24_H_25_N_3_O_5_, the eth­oxy group is disordered over two orientations in a 0.853 (14):0.147 (14) ratio. The benzimadazole ring system (r.m.s. deviation = 0.016 Å) makes a dihedral angle of 35.47 (7)° with the attached benzene ring. The pyrrolidine ring adopts an envelope conformation with a methyl­ene C atom as the flap. In the crystal, inversion dimers linked by pairs of O—H⋯N hydrogen bonds generate *R*_2_^2^(16) loops. C—H⋯O inter­actions link the dimers into a three-dimensional network.

## Related literature

For a related structure and background to benzimidazoles, see: Yoon *et al.* (2012 ▶). For the stability of the temperature controller used in the data collection, see: Cosier & Glazer (1986 ▶). For puckering parameters, see: Cremer & Pople (1975 ▶).
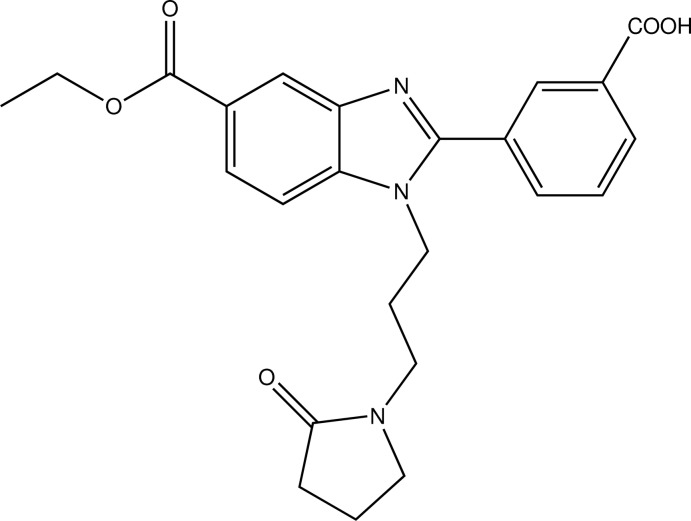


## Experimental

### 

#### Crystal data

C_24_H_25_N_3_O_5_
*M* *_r_* = 435.47
Monoclinic, 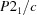

*a* = 9.3097 (3) Å
*b* = 24.8252 (7) Å
*c* = 9.6761 (3) Å
β = 112.322 (1)°
*V* = 2068.71 (11) Å^3^
*Z* = 4
Mo *K*α radiation
μ = 0.10 mm^−1^
*T* = 100 K
0.39 × 0.23 × 0.08 mm

#### Data collection

Bruker SMART APEXII CCD diffractometer
Absorption correction: multi-scan (*SADABS*; Bruker, 2009 ▶) *T*_min_ = 0.963, *T*_max_ = 0.992
23636 measured reflections
6010 independent reflections
3706 reflections with *I* > 2σ(*I*)
*R*_int_ = 0.065

#### Refinement

*R*[*F* ^2^ > 2σ(*F* ^2^)] = 0.064
*wR*(*F* ^2^) = 0.134
*S* = 1.03
6010 reflections
317 parameters
69 restraints
H atoms treated by a mixture of independent and constrained refinement
Δρ_max_ = 0.37 e Å^−3^
Δρ_min_ = −0.31 e Å^−3^

### 

Data collection: *APEX2* (Bruker, 2009 ▶); cell refinement: *SAINT* (Bruker, 2009 ▶); data reduction: *SAINT*; program(s) used to solve structure: *SHELXTL* (Sheldrick, 2008 ▶); program(s) used to refine structure: *SHELXTL*; molecular graphics: *SHELXTL*; software used to prepare material for publication: *SHELXTL* and *PLATON* (Spek, 2009 ▶).

## Supplementary Material

Crystal structure: contains datablock(s) I, global. DOI: 10.1107/S1600536813001116/hb7017sup1.cif

Structure factors: contains datablock(s) I. DOI: 10.1107/S1600536813001116/hb7017Isup2.hkl

Supplementary material file. DOI: 10.1107/S1600536813001116/hb7017Isup3.cml

Additional supplementary materials:  crystallographic information; 3D view; checkCIF report

## Figures and Tables

**Table 1:** Hydrogen-bond geometry (Å, °)

*D*—H⋯*A*	*D*—H	H⋯*A*	*D*⋯*A*	*D*—H⋯*A*
O2—H1*O*2⋯N2^i^	1.04 (4)	1.66 (4)	2.675 (2)	166 (3)
C5—H5*A*⋯O3^i^	0.95	2.57	3.367 (2)	141
C9—H9*A*⋯O2^i^	0.95	2.46	3.177 (2)	132
C15—H15*A*⋯O3^ii^	0.99	2.34	3.314 (3)	167
C17—H17*B*⋯O5^iii^	0.99	2.51	3.429 (3)	154
C20—H20*B*⋯O4^iv^	0.99	2.55	3.340 (3)	137

Symmetry codes: (i) 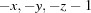; (ii) 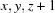; (iii) 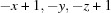; (iv) 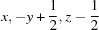.

**Fig. 1.**
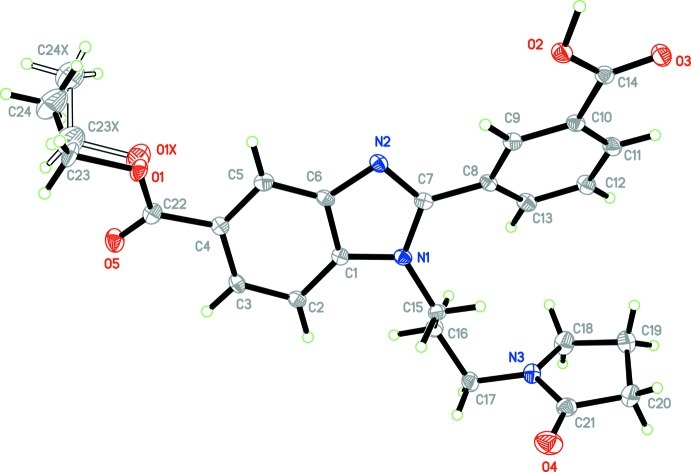
The structure of the title compound, showing 50% probability displacement ellipsoids.

**Fig. 2.**
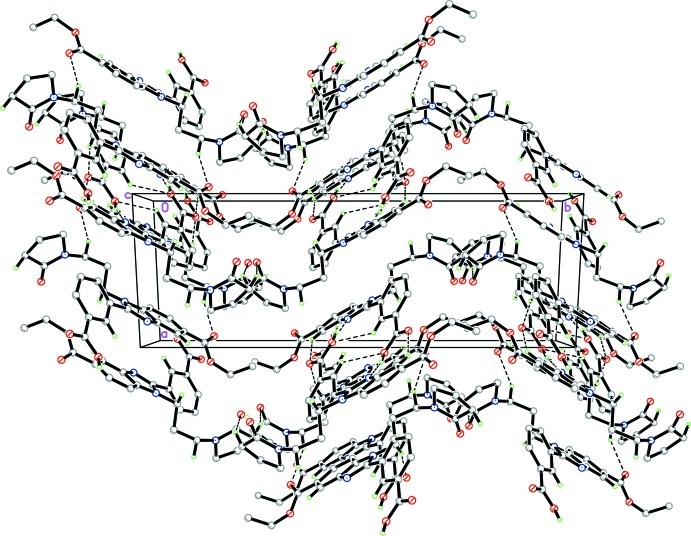
The crystal packing of (I). Dashed lines indicate hydrogen bonds. H atoms not involved in the hydrogen bond interactions have been omitted for clarity.

## supplementary crystallographic information

### Comment

As part of our ongoing structural studies of benzimidazole derivatives (Yoon *et al.*, 2012), we now report the structure of the title compound.

The ethoxy group is disordered over two position with the final refined occupancies of 0.853 (14):0.147 (14). The benzimidazole (N1—N2/C—C7) ring in the title compound, Fig. 1, is almost planar with maximum deviation of 0.02 Å for atom N2 and makes a dihedral angle of 35.63° with the attached benzene ring (C8—C13). The pyrrolidine ring (N3/C18—C21) adopts an envelope conformation with C19 as the flap and puckering parameters Q = 0.262 (3) Å and φ = 64.0 (5)°.

In the crystal (Fig. 2), O—H···N and C—H···O interactions (Table 1) link the molecules into a three-dimensional network.

### Experimental

Ethyl 3-amino-4-(3(2-oxopyrrolidin-1yl)propylamino)benzoate (0.84 mmol) and sodium metabisulfite adduct of 4-carboxy benzaldehyde (1.68 mmol) were dissolved in DMF. The reaction mixture was reflux at 130 °C for 2 hrs. After completion, the reaction mixture was diluted in Ethyl acetate (20 ml) and washed with water (20 ml). The organic layer was collected, dried over Na_2_SO_4_ and the evaporated *in vacuo* to yield the product. The product was recrystallized from ethyl acetate as brown plates.

### Refinement

O bound H atoms were located from a difference Fourier maps and freely refined. The remaining H atoms were positioned geometrically and refined using a riding model with C—H = 0.95–0.99 Å and *U*_iso_(H) = 1.2*U*_eq_(C) and 1.5*U*_eq_(*C*-methyl). The ethoxy group disordered over two postion with the final refine occupancies 0.853 (14):0.147 (14). A rigid bond and similar restraint were applied for the disordered component. The same anisotropic displacement are used for the C23X and C24X.

### Crystal data

**Table:**

C_24_H_25_N_3_O_5_	*F*(000) = 920
*M**_r_* = 435.47	*D*_x_ = 1.398 Mg m^−^^3^
Monoclinic, *P*2_1_/*c*	Mo *K*α radiation, λ = 0.71073 Å
Hall symbol: -P 2ybc	Cell parameters from 3829 reflections
*a* = 9.3097 (3) Å	θ = 2.4–29.4°
*b* = 24.8252 (7) Å	µ = 0.10 mm^−^^1^
*c* = 9.6761 (3) Å	*T* = 100 K
β = 112.322 (1)°	Plate, brown
*V* = 2068.71 (11) Å^3^	0.39 × 0.23 × 0.08 mm
*Z* = 4	

### Data collection

**Table:**

Bruker SMART APEXII CCD diffractometer	6010 independent reflections
Radiation source: fine-focus sealed tube	3706 reflections with *I* > 2σ(*I*)
Graphite monochromator	*R*_int_ = 0.065
φ and ω scans	θ_max_ = 30.1°, θ_min_ = 2.4°
Absorption correction: multi-scan (*SADABS*; Bruker, 2009)	*h* = −13→13
*T*_min_ = 0.963, *T*_max_ = 0.992	*k* = −34→34
23636 measured reflections	*l* = −8→13

### Refinement

**Table:**

Refinement on *F*^2^	Primary atom site location: structure-invariant direct methods
Least-squares matrix: full	Secondary atom site location: difference Fourier map
*R*[*F*^2^ > 2σ(*F*^2^)] = 0.064	Hydrogen site location: inferred from neighbouring sites
*wR*(*F*^2^) = 0.134	H atoms treated by a mixture of independent and constrained refinement
*S* = 1.03	*w* = 1/[σ^2^(*F*_o_^2^) + (0.0444*P*)^2^ + 0.6946*P*] where *P* = (*F*_o_^2^ + 2*F*_c_^2^)/3
6010 reflections	(Δ/σ)_max_ = 0.001
317 parameters	Δρ_max_ = 0.37 e Å^−^^3^
69 restraints	Δρ_min_ = −0.31 e Å^−^^3^

### Special details

**Table:**

Experimental. The crystal was placed in the cold stream of an Oxford Cryosystems Cobra open-flow nitrogen cryostat (Cosier & Glazer, 1986) operating at 100.0 (1) K.
Geometry. All e.s.d.'s (except the e.s.d. in the dihedral angle between two l.s. planes) are estimated using the full covariance matrix. The cell e.s.d.'s are taken into account individually in the estimation of e.s.d.'s in distances, angles and torsion angles; correlations between e.s.d.'s in cell parameters are only used when they are defined by crystal symmetry. An approximate (isotropic) treatment of cell e.s.d.'s is used for estimating e.s.d.'s involving l.s. planes.
Refinement. Refinement of *F*^2^ against ALL reflections. The weighted *R*-factor *wR* and goodness of fit *S* are based on *F*^2^, conventional *R*-factors *R* are based on *F*, with *F* set to zero for negative *F*^2^. The threshold expression of *F*^2^ > σ(*F*^2^) is used only for calculating *R*-factors(gt) *etc*. and is not relevant to the choice of reflections for refinement. *R*-factors based on *F*^2^ are statistically about twice as large as those based on *F*, and *R*- factors based on ALL data will be even larger.

### Fractional atomic coordinates and isotropic or equivalent isotropic displacement parameters (Å^2^)

**Table:**

	*x*	*y*	*z*	*U*_iso_*/*U*_eq_	Occ. (<1)
O1	−0.1036 (4)	−0.15612 (14)	0.1235 (6)	0.0179 (7)	0.853 (14)
C23	−0.1927 (4)	−0.20029 (16)	0.1526 (4)	0.0216 (8)	0.853 (14)
H23A	−0.3036	−0.1964	0.0868	0.026*	0.853 (14)
H23B	−0.1847	−0.1983	0.2575	0.026*	0.853 (14)
C24	−0.1357 (4)	−0.25307 (15)	0.1261 (8)	0.0427 (14)	0.853 (14)
H24A	−0.1986	−0.2817	0.1448	0.064*	0.853 (14)
H24B	−0.0270	−0.2575	0.1935	0.064*	0.853 (14)
H24C	−0.1437	−0.2551	0.0223	0.064*	0.853 (14)
O1X	−0.066 (4)	−0.1693 (10)	0.123 (4)	0.043 (6)	0.147 (14)
C23X	−0.148 (5)	−0.2161 (11)	0.153 (3)	0.054 (5)	0.147 (14)
H23C	−0.1018	−0.2268	0.2591	0.064*	0.147 (14)
H23D	−0.2598	−0.2080	0.1259	0.064*	0.147 (14)
C24X	−0.127 (4)	−0.2575 (9)	0.057 (3)	0.054 (5)	0.147 (14)
H24D	−0.1522	−0.2927	0.0879	0.080*	0.147 (14)
H24E	−0.0185	−0.2575	0.0657	0.080*	0.147 (14)
H24F	−0.1952	−0.2504	−0.0466	0.080*	0.147 (14)
O2	−0.02946 (16)	0.06006 (5)	−0.61398 (17)	0.0181 (3)	
O3	0.12028 (16)	0.11302 (5)	−0.69174 (16)	0.0201 (3)	
O4	0.43579 (19)	0.23361 (6)	0.23971 (19)	0.0333 (4)	
O5	0.06150 (17)	−0.15773 (6)	0.36566 (17)	0.0231 (3)	
N1	0.30457 (18)	0.03432 (6)	0.07554 (19)	0.0142 (3)	
N2	0.17804 (18)	−0.02457 (6)	−0.10670 (19)	0.0147 (4)	
N3	0.61523 (19)	0.17298 (6)	0.2256 (2)	0.0184 (4)	
C1	0.2460 (2)	−0.00527 (7)	0.1413 (2)	0.0135 (4)	
C2	0.2562 (2)	−0.01238 (8)	0.2868 (2)	0.0145 (4)	
H2A	0.3106	0.0124	0.3638	0.017*	
C3	0.1832 (2)	−0.05727 (7)	0.3144 (2)	0.0151 (4)	
H3A	0.1897	−0.0640	0.4133	0.018*	
C4	0.0994 (2)	−0.09329 (7)	0.1994 (2)	0.0147 (4)	
C5	0.0890 (2)	−0.08568 (8)	0.0537 (2)	0.0158 (4)	
H5A	0.0315	−0.1097	−0.0240	0.019*	
C6	0.1663 (2)	−0.04131 (7)	0.0261 (2)	0.0139 (4)	
C7	0.2601 (2)	0.02085 (7)	−0.0722 (2)	0.0139 (4)	
C8	0.2977 (2)	0.05271 (7)	−0.1826 (2)	0.0145 (4)	
C9	0.1876 (2)	0.05557 (7)	−0.3289 (2)	0.0148 (4)	
H9A	0.0924	0.0365	−0.3548	0.018*	
C10	0.2155 (2)	0.08584 (7)	−0.4364 (2)	0.0148 (4)	
C11	0.3555 (2)	0.11325 (8)	−0.3997 (2)	0.0178 (4)	
H11A	0.3739	0.1347	−0.4725	0.021*	
C12	0.4683 (2)	0.10921 (8)	−0.2565 (2)	0.0185 (4)	
H12A	0.5650	0.1271	−0.2325	0.022*	
C13	0.4402 (2)	0.07915 (8)	−0.1481 (2)	0.0177 (4)	
H13A	0.5179	0.0765	−0.0504	0.021*	
C14	0.0980 (2)	0.08844 (7)	−0.5930 (2)	0.0153 (4)	
C15	0.3717 (2)	0.08531 (7)	0.1508 (2)	0.0155 (4)	
H15A	0.3073	0.0989	0.2044	0.019*	
H15B	0.3679	0.1123	0.0742	0.019*	
C16	0.5390 (2)	0.07964 (8)	0.2613 (2)	0.0176 (4)	
H16A	0.6042	0.0656	0.2090	0.021*	
H16B	0.5435	0.0536	0.3404	0.021*	
C17	0.6019 (3)	0.13412 (8)	0.3320 (2)	0.0212 (5)	
H17A	0.5322	0.1489	0.3786	0.025*	
H17B	0.7054	0.1287	0.4121	0.025*	
C18	0.7451 (3)	0.17112 (9)	0.1760 (3)	0.0262 (5)	
H18A	0.7494	0.1360	0.1293	0.031*	
H18B	0.8451	0.1774	0.2604	0.031*	
C19	0.7087 (3)	0.21703 (9)	0.0613 (3)	0.0283 (5)	
H19A	0.8051	0.2348	0.0645	0.034*	
H19B	0.6505	0.2036	−0.0411	0.034*	
C20	0.6095 (3)	0.25556 (8)	0.1106 (3)	0.0280 (5)	
H20A	0.6740	0.2846	0.1743	0.034*	
H20B	0.5269	0.2720	0.0230	0.034*	
C21	0.5400 (2)	0.22093 (8)	0.1978 (2)	0.0206 (5)	
C22	0.0213 (2)	−0.13958 (8)	0.2405 (2)	0.0181 (4)	
H1O2	−0.085 (4)	0.0523 (13)	−0.727 (4)	0.088 (12)*	

### Atomic displacement parameters (Å^2^)

**Table:**

	*U*^11^	*U*^22^	*U*^33^	*U*^12^	*U*^13^	*U*^23^
O1	0.0142 (12)	0.0183 (13)	0.0183 (12)	−0.0053 (9)	0.0027 (10)	0.0015 (11)
C23	0.0192 (16)	0.0166 (16)	0.0258 (16)	−0.0079 (11)	0.0050 (13)	0.0021 (12)
C24	0.0372 (19)	0.0253 (16)	0.064 (4)	−0.0002 (13)	0.018 (2)	−0.0081 (19)
O1X	0.062 (15)	0.034 (9)	0.021 (8)	−0.021 (9)	0.004 (11)	0.003 (9)
C23X	0.090 (13)	0.025 (8)	0.054 (10)	−0.008 (8)	0.037 (10)	−0.004 (8)
C24X	0.090 (13)	0.025 (8)	0.054 (10)	−0.008 (8)	0.037 (10)	−0.004 (8)
O2	0.0158 (7)	0.0229 (7)	0.0141 (8)	−0.0050 (6)	0.0041 (6)	−0.0017 (6)
O3	0.0208 (7)	0.0225 (7)	0.0168 (8)	−0.0016 (6)	0.0069 (6)	0.0022 (6)
O4	0.0335 (9)	0.0364 (9)	0.0320 (11)	0.0089 (7)	0.0148 (8)	−0.0014 (8)
O5	0.0236 (8)	0.0253 (8)	0.0175 (8)	−0.0030 (6)	0.0047 (7)	0.0059 (6)
N1	0.0123 (8)	0.0176 (8)	0.0125 (9)	−0.0010 (6)	0.0045 (7)	−0.0019 (7)
N2	0.0119 (8)	0.0194 (8)	0.0115 (9)	−0.0008 (6)	0.0029 (7)	0.0002 (7)
N3	0.0173 (9)	0.0187 (8)	0.0185 (10)	−0.0009 (7)	0.0060 (8)	0.0018 (7)
C1	0.0100 (9)	0.0159 (9)	0.0143 (10)	0.0005 (7)	0.0042 (8)	−0.0003 (8)
C2	0.0134 (9)	0.0174 (9)	0.0113 (10)	0.0008 (7)	0.0031 (8)	0.0002 (7)
C3	0.0132 (9)	0.0192 (10)	0.0132 (10)	0.0017 (8)	0.0053 (8)	0.0028 (8)
C4	0.0124 (9)	0.0169 (9)	0.0144 (10)	0.0010 (7)	0.0046 (8)	0.0011 (8)
C5	0.0120 (9)	0.0178 (9)	0.0159 (11)	0.0001 (7)	0.0033 (8)	−0.0019 (8)
C6	0.0122 (9)	0.0183 (9)	0.0097 (10)	0.0015 (7)	0.0024 (8)	0.0003 (8)
C7	0.0105 (9)	0.0166 (9)	0.0128 (10)	0.0021 (7)	0.0026 (8)	−0.0002 (8)
C8	0.0146 (10)	0.0172 (9)	0.0130 (10)	0.0014 (7)	0.0067 (8)	−0.0001 (8)
C9	0.0125 (9)	0.0162 (9)	0.0156 (11)	−0.0004 (7)	0.0052 (8)	−0.0014 (8)
C10	0.0154 (9)	0.0154 (9)	0.0139 (10)	0.0018 (7)	0.0058 (8)	−0.0010 (8)
C11	0.0178 (10)	0.0206 (10)	0.0177 (11)	−0.0034 (8)	0.0096 (9)	−0.0018 (8)
C12	0.0151 (10)	0.0234 (10)	0.0180 (11)	−0.0038 (8)	0.0076 (9)	−0.0011 (9)
C13	0.0145 (10)	0.0223 (10)	0.0145 (11)	−0.0004 (8)	0.0036 (9)	−0.0009 (8)
C14	0.0160 (10)	0.0154 (9)	0.0152 (11)	0.0010 (8)	0.0066 (9)	−0.0007 (8)
C15	0.0168 (10)	0.0145 (9)	0.0158 (11)	−0.0007 (8)	0.0069 (9)	−0.0024 (8)
C16	0.0175 (10)	0.0176 (10)	0.0149 (11)	−0.0006 (8)	0.0032 (9)	0.0007 (8)
C17	0.0256 (11)	0.0217 (10)	0.0130 (11)	−0.0055 (9)	0.0037 (9)	0.0013 (8)
C18	0.0201 (11)	0.0253 (11)	0.0361 (15)	0.0011 (9)	0.0141 (11)	0.0038 (10)
C19	0.0347 (13)	0.0267 (11)	0.0276 (13)	−0.0046 (10)	0.0165 (11)	0.0015 (10)
C20	0.0372 (14)	0.0202 (11)	0.0266 (13)	−0.0011 (10)	0.0120 (11)	0.0029 (9)
C21	0.0219 (11)	0.0209 (10)	0.0169 (11)	0.0007 (8)	0.0048 (9)	−0.0016 (8)
C22	0.0143 (10)	0.0172 (9)	0.0216 (12)	0.0001 (8)	0.0056 (9)	0.0005 (9)

### Geometric parameters (Å, º)

**Table:**

O1—C22	1.343 (5)	C3—H3A	0.9500
O1—C23	1.465 (3)	C4—C5	1.388 (3)
C23—C24	1.472 (4)	C4—C22	1.492 (3)
C23—H23A	0.9900	C5—C6	1.396 (3)
C23—H23B	0.9900	C5—H5A	0.9500
C24—H24A	0.9800	C7—C8	1.475 (3)
C24—H24B	0.9800	C8—C9	1.399 (3)
C24—H24C	0.9800	C8—C13	1.402 (3)
O1X—C22	1.34 (3)	C9—C10	1.386 (3)
O1X—C23X	1.478 (17)	C9—H9A	0.9500
C23X—C24X	1.446 (17)	C10—C11	1.392 (3)
C23X—H23C	0.9900	C10—C14	1.495 (3)
C23X—H23D	0.9900	C11—C12	1.388 (3)
C24X—H24D	0.9800	C11—H11A	0.9500
C24X—H24E	0.9800	C12—C13	1.390 (3)
C24X—H24F	0.9800	C12—H12A	0.9500
O2—C14	1.328 (2)	C13—H13A	0.9500
O2—H1O2	1.03 (4)	C15—C16	1.525 (3)
O3—C14	1.216 (2)	C15—H15A	0.9900
O4—C21	1.226 (2)	C15—H15B	0.9900
O5—C22	1.211 (2)	C16—C17	1.528 (3)
N1—C7	1.370 (2)	C16—H16A	0.9900
N1—C1	1.390 (2)	C16—H16B	0.9900
N1—C15	1.475 (2)	C17—H17A	0.9900
N2—C7	1.331 (2)	C17—H17B	0.9900
N2—C6	1.394 (2)	C18—C19	1.536 (3)
N3—C21	1.355 (3)	C18—H18A	0.9900
N3—C17	1.450 (3)	C18—H18B	0.9900
N3—C18	1.462 (3)	C19—C20	1.527 (3)
C1—C2	1.386 (3)	C19—H19A	0.9900
C1—C6	1.401 (3)	C19—H19B	0.9900
C2—C3	1.383 (3)	C20—C21	1.511 (3)
C2—H2A	0.9500	C20—H20A	0.9900
C3—C4	1.410 (3)	C20—H20B	0.9900
			
C22—O1—C23	116.1 (4)	C12—C11—C10	119.99 (19)
O1—C23—C24	111.4 (3)	C12—C11—H11A	120.0
O1—C23—H23A	109.3	C10—C11—H11A	120.0
C24—C23—H23A	109.3	C11—C12—C13	120.27 (19)
O1—C23—H23B	109.3	C11—C12—H12A	119.9
C24—C23—H23B	109.3	C13—C12—H12A	119.9
H23A—C23—H23B	108.0	C12—C13—C8	120.24 (19)
C22—O1X—C23X	117 (2)	C12—C13—H13A	119.9
C24X—C23X—O1X	103.0 (18)	C8—C13—H13A	119.9
C24X—C23X—H23C	111.2	O3—C14—O2	123.72 (19)
O1X—C23X—H23C	111.2	O3—C14—C10	122.72 (18)
C24X—C23X—H23D	111.2	O2—C14—C10	113.53 (17)
O1X—C23X—H23D	111.2	N1—C15—C16	113.28 (15)
H23C—C23X—H23D	109.1	N1—C15—H15A	108.9
C23X—C24X—H24D	109.5	C16—C15—H15A	108.9
C23X—C24X—H24E	109.5	N1—C15—H15B	108.9
H24D—C24X—H24E	109.5	C16—C15—H15B	108.9
C23X—C24X—H24F	109.5	H15A—C15—H15B	107.7
H24D—C24X—H24F	109.5	C15—C16—C17	110.36 (16)
H24E—C24X—H24F	109.5	C15—C16—H16A	109.6
C14—O2—H1O2	108.8 (19)	C17—C16—H16A	109.6
C7—N1—C1	106.81 (15)	C15—C16—H16B	109.6
C7—N1—C15	128.88 (16)	C17—C16—H16B	109.6
C1—N1—C15	123.18 (16)	H16A—C16—H16B	108.1
C7—N2—C6	105.04 (16)	N3—C17—C16	113.13 (17)
C21—N3—C17	123.25 (18)	N3—C17—H17A	109.0
C21—N3—C18	113.08 (17)	C16—C17—H17A	109.0
C17—N3—C18	121.13 (17)	N3—C17—H17B	109.0
C2—C1—N1	131.88 (18)	C16—C17—H17B	109.0
C2—C1—C6	122.52 (18)	H17A—C17—H17B	107.8
N1—C1—C6	105.60 (17)	N3—C18—C19	103.34 (17)
C3—C2—C1	116.74 (18)	N3—C18—H18A	111.1
C3—C2—H2A	121.6	C19—C18—H18A	111.1
C1—C2—H2A	121.6	N3—C18—H18B	111.1
C2—C3—C4	121.57 (19)	C19—C18—H18B	111.1
C2—C3—H3A	119.2	H18A—C18—H18B	109.1
C4—C3—H3A	119.2	C20—C19—C18	103.47 (18)
C5—C4—C3	121.29 (18)	C20—C19—H19A	111.1
C5—C4—C22	121.47 (18)	C18—C19—H19A	111.1
C3—C4—C22	117.23 (18)	C20—C19—H19B	111.1
C4—C5—C6	117.34 (18)	C18—C19—H19B	111.1
C4—C5—H5A	121.3	H19A—C19—H19B	109.0
C6—C5—H5A	121.3	C21—C20—C19	104.83 (17)
N2—C6—C5	129.66 (18)	C21—C20—H20A	110.8
N2—C6—C1	109.85 (17)	C19—C20—H20A	110.8
C5—C6—C1	120.48 (19)	C21—C20—H20B	110.8
N2—C7—N1	112.70 (17)	C19—C20—H20B	110.8
N2—C7—C8	122.94 (18)	H20A—C20—H20B	108.9
N1—C7—C8	124.36 (17)	O4—C21—N3	125.0 (2)
C9—C8—C13	118.74 (18)	O4—C21—C20	126.77 (19)
C9—C8—C7	118.40 (17)	N3—C21—C20	108.20 (18)
C13—C8—C7	122.84 (19)	O5—C22—O1X	120.1 (11)
C10—C9—C8	120.84 (18)	O5—C22—O1	124.7 (2)
C10—C9—H9A	119.6	O1X—C22—O1	20.7 (16)
C8—C9—H9A	119.6	O5—C22—C4	123.81 (19)
C9—C10—C11	119.84 (19)	O1X—C22—C4	113.4 (11)
C9—C10—C14	120.71 (17)	O1—C22—C4	111.4 (2)
C11—C10—C14	119.43 (18)		
			
C22—O1—C23—C24	−90.3 (5)	C10—C11—C12—C13	−1.9 (3)
C22—O1X—C23X—C24X	−138 (3)	C11—C12—C13—C8	0.0 (3)
C7—N1—C1—C2	179.4 (2)	C9—C8—C13—C12	2.4 (3)
C15—N1—C1—C2	−11.8 (3)	C7—C8—C13—C12	−179.14 (18)
C7—N1—C1—C6	−0.3 (2)	C9—C10—C14—O3	−177.43 (18)
C15—N1—C1—C6	168.44 (16)	C11—C10—C14—O3	1.2 (3)
N1—C1—C2—C3	−179.75 (19)	C9—C10—C14—O2	0.5 (3)
C6—C1—C2—C3	0.0 (3)	C11—C10—C14—O2	179.16 (17)
C1—C2—C3—C4	−1.5 (3)	C7—N1—C15—C16	−113.5 (2)
C2—C3—C4—C5	1.2 (3)	C1—N1—C15—C16	80.4 (2)
C2—C3—C4—C22	−178.28 (18)	N1—C15—C16—C17	178.68 (17)
C3—C4—C5—C6	0.8 (3)	C21—N3—C17—C16	120.6 (2)
C22—C4—C5—C6	−179.84 (17)	C18—N3—C17—C16	−78.7 (2)
C7—N2—C6—C5	178.0 (2)	C15—C16—C17—N3	−65.6 (2)
C7—N2—C6—C1	−1.1 (2)	C21—N3—C18—C19	−20.0 (2)
C4—C5—C6—N2	178.75 (18)	C17—N3—C18—C19	177.57 (18)
C4—C5—C6—C1	−2.2 (3)	N3—C18—C19—C20	26.0 (2)
C2—C1—C6—N2	−178.87 (17)	C18—C19—C20—C21	−23.6 (2)
N1—C1—C6—N2	0.9 (2)	C17—N3—C21—O4	−11.8 (3)
C2—C1—C6—C5	2.0 (3)	C18—N3—C21—O4	−173.9 (2)
N1—C1—C6—C5	−178.27 (17)	C17—N3—C21—C20	166.91 (18)
C6—N2—C7—N1	0.9 (2)	C18—N3—C21—C20	4.9 (2)
C6—N2—C7—C8	−179.02 (17)	C19—C20—C21—O4	−168.8 (2)
C1—N1—C7—N2	−0.4 (2)	C19—C20—C21—N3	12.5 (2)
C15—N1—C7—N2	−168.29 (17)	C23X—O1X—C22—O5	18 (3)
C1—N1—C7—C8	179.56 (17)	C23X—O1X—C22—O1	−91 (4)
C15—N1—C7—C8	11.6 (3)	C23X—O1X—C22—C4	−179.9 (15)
N2—C7—C8—C9	35.6 (3)	C23—O1—C22—O5	−1.6 (4)
N1—C7—C8—C9	−144.28 (19)	C23—O1—C22—O1X	82 (4)
N2—C7—C8—C13	−142.87 (19)	C23—O1—C22—C4	−177.9 (2)
N1—C7—C8—C13	37.2 (3)	C5—C4—C22—O5	156.8 (2)
C13—C8—C9—C10	−2.8 (3)	C3—C4—C22—O5	−23.7 (3)
C7—C8—C9—C10	178.65 (17)	C5—C4—C22—O1X	−4.5 (18)
C8—C9—C10—C11	0.9 (3)	C3—C4—C22—O1X	174.9 (18)
C8—C9—C10—C14	179.48 (17)	C5—C4—C22—O1	−26.8 (3)
C9—C10—C11—C12	1.5 (3)	C3—C4—C22—O1	152.6 (2)
C14—C10—C11—C12	−177.12 (18)		

### Hydrogen-bond geometry (Å, º)

**Table:**

*D*—H···*A*	*D*—H	H···*A*	*D*···*A*	*D*—H···*A*
O2—H1*O*2···N2^i^	1.04 (4)	1.66 (4)	2.675 (2)	166 (3)
C5—H5*A*···O3^i^	0.95	2.57	3.367 (2)	141
C9—H9*A*···O2^i^	0.95	2.46	3.177 (2)	132
C15—H15*A*···O3^ii^	0.99	2.34	3.314 (3)	167
C17—H17*B*···O5^iii^	0.99	2.51	3.429 (3)	154
C20—H20*B*···O4^iv^	0.99	2.55	3.340 (3)	137

Symmetry codes: (i) −*x*, −*y*, −*z*−1; (ii) *x*, *y*, *z*+1; (iii) −*x*+1, −*y*, −*z*+1; (iv) *x*, −*y*+1/2, *z*−1/2.

## References

[bb1] Bruker (2009). *APEX2*, *SAINT* and *SADABS* Bruker AXS Inc., Madison, Wisconsin, USA.

[bb2] Cosier, J. & Glazer, A. M. (1986). *J. Appl. Cryst.* **19**, 105–107.

[bb3] Cremer, D. & Pople, J. A. (1975). *J. Am. Chem. Soc.* **97**, 1354–1358.

[bb4] Sheldrick, G. M. (2008). *Acta Cryst.* A**64**, 112–122. 10.1107/S0108767307043930 18156677

[bb5] Spek, A. L. (2009). *Acta Cryst.* D**65**, 148–155. 10.1107/S090744490804362X PMC2631630 19171970

[bb6] Yoon, Y. K., Ali, M. A., Choon, T. S., Asik, S. I. J. & Razak, I. A. (2012). *Acta Cryst.* E**68**, o59. 10.1107/S1600536811052408 PMC3254415 22259559

